# Comparison of post-treatment changes with and without retention in adolescents treated for maxillary impacted canines—a randomized controlled trial

**DOI:** 10.1093/ejo/cjaa010

**Published:** 2020-03-05

**Authors:** Sasan Naraghi, Niels Ganzer, Lars Bondemark, Mikael Sonesson

**Affiliations:** 1 Orthodontic Clinic, National Health Service, Växjö, Sweden; 2 Department of Orthodontics, University of Malmö, Sweden; 3 Orthodontic Clinic, Public Dental Health, Gävle, Sweden; 4 Centre for Research and Development Uppsala University/Region Gävleborg, Gävle, Sweden

## Abstract

**Objective:**

To evaluate whether retention is needed after orthodontic treatment of impacted maxillary canines.

**Trial design:**

Two-arm parallel group single-centre randomized controlled trial.

**Materials and methods:**

Sixty-three patients, 39 girls and 24 boys, were recruited to the study. The inclusion criteria were patients with at least one impacted or unerupted maxillary canine, and moderate irregularity of the maxillary six anterior teeth according to Little’s index (LI). After gaining informed consent from the patient and their custodians, the patients were randomized to one of two groups, i.e. to a non-retention group or a retention group. The randomization process was prepared and carried out by an independent person not involved in the trial and the randomization used blocks of 20 (10 + 10). Primary outcomes were changes in single contact point discrepancy, and LI measured on digitalized three-dimensional study casts 1-year post-treatment. The study casts were anonymized before assessment and the changes were blinded for the assessor. Data were evaluated on an intention-to-treat basis. Thus, all randomized patients were incorporated into the final analysis. In the non-retention group a 10-week interim period was used to detect patients who eventually have a relapse immediately after treatment. If so, the patient got the arch-wire reinserted. Most patients in the retention group received a vacuum-formed retainer and pretreatment spacing cases got a bonded retainer.

**Results:**

Mean irregularity change was 0.4 mm in the retention and 1.3 mm in the non-retention group (*P* < 0.001). Maximum change was 2.5 mm in the retention and 3.2 mm in the non-retention group (*P* < 0.001). Most changes in the non-retention group occurred during the 10-week interim period. In the non-retention group, one patient developed contact point discrepancy of >2 mm during the interim period and was realigned.

**Harms:**

One patient met the stopping guideline criteria. This patient had the arch wire reinserted for 2 months. After realignment, the patient received a retention appliance.

**Limitations:**

The trial was a single-centre study and short-term changes were evaluated.

**Conclusions:**

Changes between the retention and the non-retention group were statistically but not clinically significant. Since satisfactory clinical results 1-year post-treatment were found in the non-retention group, retention does not appear to be needed. The 10-week interim period was useful in detecting patients who might have a relapse immediately after treatment.

**Trial registration:**

The trial was not registered.

## Introduction

Aesthetics, and in particular the aesthetics of maxillary anterior teeth, are the primary reason for patients seeking orthodontic treatment (1). Therefore, the stability of the maxillary anterior teeth is crucial for both the patients and the orthodontist (2–5). Since retention is one of the most challenging problems in orthodontics, every orthodontist has a philosophy, strategy, and strong belief as to how retention should be provided (6, 7). This belief is rarely evidence-based but instead usually based on clinical experience. The dominant opinion is to keep the retention for a very long time and in many cases life-long retention is recommended (8, 9).

It has been shown that relapse occurs independently of retention methods. Removable retainers are dependent on the compliance of the patients and bonded retainers are exposed to wire fractures or composite breakages (10–13). Furthermore, difficulties in cleaning approximal surfaces might increase the prevalence of periodontal complications (14, 15). Thus, it may be desirable to identify patients who do not need any retention.

Previous studies on retention and mandibular anterior teeth show promising results in patients treated solely by interproximal enamel reduction (IPR) (16–18). However, trials on patients without any retention or IPR do not appear to exist.

Maxillary anterior teeth have been presented as being more stable to post-treatment changes compared to mandibular teeth (19–22). Moreover, among the six maxillary anterior teeth, the canines were reported to be more stable than the incisors (23, 24). This implies that patients with reasonably straight maxillary incisors before treatment potentially do not need any retention. As the incidence of maxillary canine impaction is between 1.8 and 3.3 per cent (25, 26), a considerable number of patients must be treated. These patients might benefit from an improved retention regimen. Therefore, the purpose of this trial was to evaluate whether retention is needed after orthodontic treatment for impacted maxillary canines and with moderate pre-treatment irregularity in the maxilla. We hypothesized that no statistically or clinically significant difference will occur in the position of the maxillary anterior teeth of patients with and without retention, 1-year post-treatment.

## Subjects and methods

### Trial design

The trial was a single-centre randomized controlled trial (RCT) with two parallel arms and a 1:1 allocation ratio. The primary outcome was post-treatment changes in irregularity of the maxillary six anterior teeth in patients with and without retention. The trial period lasted between the removal of the fixed orthodontic appliances (T1) and 1 year after debond (T2). The Regional Ethical Research Board, Linköping, Sweden, who follows the Declaration of Helsinki, approved the trial (Dnr 2013/130).

### Participants

All participants were recruited from patients referred to the Orthodontic Clinic in Växjö, Public Dental Service, Kronoberg County, Sweden.

The inclusion criteria were patients who had at least one impacted or unerupted maxillary canine; and moderate irregularity of the maxillary six anterior teeth of 4–6 mm according to Little’s index (LI) (27).

The exclusion criteria were patients with agenesis or extracted maxillary anterior teeth; patients with rotations exceeding 45 degrees of one of the maxillary incisors; patients in need of orthognathic surgery; and patients with syndromes.

### Randomization

After gaining informed consent from the patient and their custodians, the patients were randomized to one of two groups, i.e. to a non-retention group or a retention group. The randomization process was prepared and carried out by an independent person not involved in the trial and the randomization used blocks of 20 (10 + 10). Opaque envelopes contained 20 sealed notes each (10 notes signed retention, and 10 notes signed non-retention). Every new participant in the trial took a note from the first envelope. When the first envelope was empty, the next envelope was opened, and so on, until the number of participants met the estimated sample size.

### Intention-to-treat

Data on all participants were evaluated on an intention-to-treat (ITT) basis. Consequently, all patients that were randomized remained in the allocated group. Patients with discontinued observation or lost to follow-up were regarded as treatment failure. Hence, the group’s maximum value for change in the primary outcome (irregularity of the maxillary six anterior teeth) as well as for the secondary variables representing the change in arch length, intercanine width and the molar width was recorded.

### Blinding

Because of the type of treatment, neither participant nor operator was blinded for the allocation. The study casts were anonymized before assessment, as was the changes of the maxillary anterior teeth for the assessor, who was not involved in the trial.

### Clinical procedure

After registration of the pre-treatment records (T0), all patients were treated by one experienced orthodontist (SN) and with pre-adjusted fixed appliance in the maxilla and mandible (0.022 slot size, MBT prescription, Victory Series, 3M Unitek, Monrovia, California, USA). After insertion of the fixed appliance and if the canine did not spontaneously erupt, the patient was referred for surgical exposure of the impacted canine. During surgical exposure, a gold chain was bonded to the impacted canine, which was then moved by the appliance to its correct place in the dental arch. After the active treatment, post-treatment records were taken (T1). The two groups were followed up 1 year after the end of active treatment when further study casts were taken (T2).

Following an interim period of 10 weeks, an analysis was performed for each patient in the non-retention group. Thus, at the end of the active treatment, the arch-wire was removed but the brackets were left in place. Then, if the orthodontist observed no changes after the 10-week period, the brackets were removed. However, if an increase in LI corresponding to greater than 3 mm or a single contact point discrepancy (CPD) of ≥2 mm was measured during the interim period, the patient continued treatment with a reinserted arch-wire. After that, a removable vacuum-formed retainer (Essix™, Erkodur, 1.5 mm 120 ø, Erkodent® Erich Kopp GmbH, Pfalzgrafenweiler, Germany) was used as a retention appliance and such patients were counted as a failure in the trial analysis.

### Retention procedure

Patients in the retention group were fitted with the upper removable vacuum-formed retainer on the day of debond and were instructed to wear the retainer 22–24 hours/day until the first visit after 4 weeks. Following that, patients were instructed to wear the vacuum-formed retainer for 10–12 hours/day. In case of pre-treatment spacing, the vacuum-formed retainer was replaced by a bonded retainer to all six teeth from canine to canine (Penta-One 0.0195, Masel, Carlsbad, California, USA).

### Outcomes

The primary outcomes were changes in single CPD and the sum of five CPDs (LI) of the maxillary six anterior teeth and measured on digitalized three-dimensional (3D) study casts between the non-retention and retention group before treatment (T0), at the end of active treatment (T1) and at the 1-year follow-up (T2).

**Figure 1.**
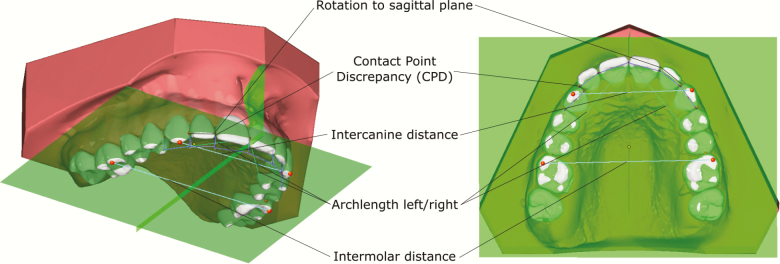
Primary and secondary outcome measures in OnyxCeph3™.

Before assessment of measurements, the study casts were digitized with a stationary 3D scanner (D700, 3Shape, Copenhagen, Denmark). On the digital models, the measurement points were located using the OnyxCeph^3^™ software (v3.2, Image Instruments, Chemnitz, Germany) with semi-automatic segmentation. The measurement points were then manually adjusted in order to improve consistency. The CPD was measured as a projection on the occlusal plane (Figure 1).

The secondary outcomes were:

- Change in arch length defined as the sum of the distances between the mesial contact point of the first permanent molar and the incisal contact point.
- Change in intercanine width, defined as the distance between the maxillary canines’ cusp tips.
- Change in intermolar width, defined as the distance between the mesiobuccal cusp tips of the maxillary first permanent molars.
- Change in the rotations of the six maxillary anterior teeth, defined as the angle between the line from the distal to the mesial contact point and the sagittal plane (Figure 1).

### Statistical analysis

The sample size calculation was based on a clinically relevant difference in LI of 3 mm (SD = 3 mm) between the groups. The level of significance was set to 5 per cent and the power to 90 per cent. The calculation resulted in a sample size of 23 patients in each group. To compensate for dropouts, at least 30 patients were planned to be enrolled in each group.

Statistical analysis was conducted using the programming language ‘R’ (v 3.60) (28). For descriptive statistics, means and standard deviations were calculated. A Shapiro–Wilk test was used for normality testing. Homogeneity of variance was tested with Levene’s modified test. Hypothesis testing was conducted with *t*-test for independent variables with a normal distribution. Independent variables that were not normally distributed were evaluated with the Mann–Whitney *U*-test. Correlations between post-treatment increase of irregularity and possible predictors were assessed with scatterplots and Spearman’s rank correlation coefficient.

### Method error analysis

Forty-five randomly selected study casts were repeatedly measured on two separate occasions with at least 2 weeks’ interval by the same examiner. Paired *t*-test revealed no significant mean differences between the two series of record occasions. *The size of the method error was determined using* Dahlberg’s formula (29). The mean measurement error for the CPDs was 0.1 mm, for LI 0.2 mm, for intercanine and intermolar width 0.2 mm each, and 2.6 degrees for tooth rotations.

## Results

### Sample characteristics

Seventy patients matching the inclusion criteria were asked to participate in this trial and, of these, 63 patients (39 females and 24 males) with a mean age of 12.9 years were accepted for participation. Enrolment commenced in June 2013 and ended in April 2018. The last follow-up (T2) was completed in April 2019. Table 1 shows the baseline demographic data.

**Table 1.** Baseline demographic data. SD, standard deviation.

Group	Gender	*n*	Age, years Mean (SD)	Impacted palatal canines, *n*	Impacted buccal canines, *n*
Retention	Female	24	13.1 (1.7)	17	10
	Male	8	12.1 (1.4)	6	2
	Total	32	12.8 (1.7)	23	12
No retention	Female	15	12.9 (1.1)	14	3
	Male	16	13.0 (1.4)	15	4
	Total	31	12.9 (1.2)	29	7
Both groups	Total	63	12.9 (1.5)	52	19

The mean duration of treatment was 29.7 (SD = 7.0) months in the retention group and 30.1 (SD = 9.1) months in the non-retention group. No significant differences in treatment duration were found between the groups or between the genders. Surgical exposure was carried out in 15 patients in the retention group and 19 patients in the non-retention group. Extraction of maxillary first premolar teeth was necessary for three patients before referral to the orthodontist in the retention group and one patient in the non-retention group. No significant differences were found between the two groups considering buccally or palatally impacted canines, angulation of canines as well as in surgical exposure of canines (Table 1).

In the retention group, 28 patients received a removable vacuum-formed retainer, and four patients received bonded retainers to all teeth canine-to-canine due to pre-treatment spacing.

**Figure 2.**
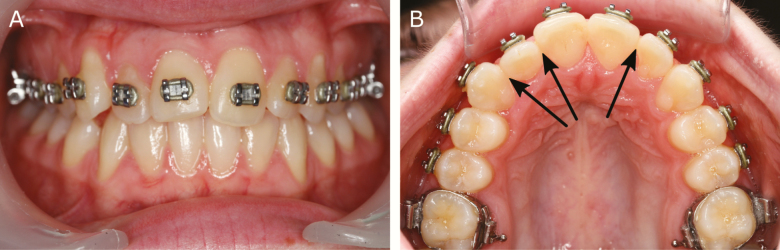
A patient who met the stopping guideline criteria during the 10-week interim period.

**Figure 3.**
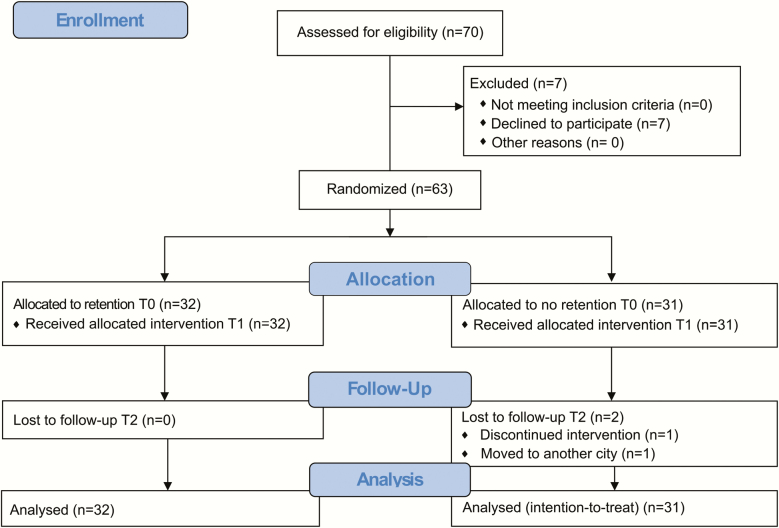
CONSORT flow chart.

In the non-retention group, one patient showed a CPD of 2 mm (Figure 2) during the 10-week interim period. According to the stopping guideline criteria, the observation period was discontinued, and the teeth were realigned. The patient received a retention appliance to avoid further relapse. Another patient in the non-retention group moved away. These two patients were considered as treatment failures in the final analysis (Figure 3).

### Changes in irregularity index

**Figure 4.**
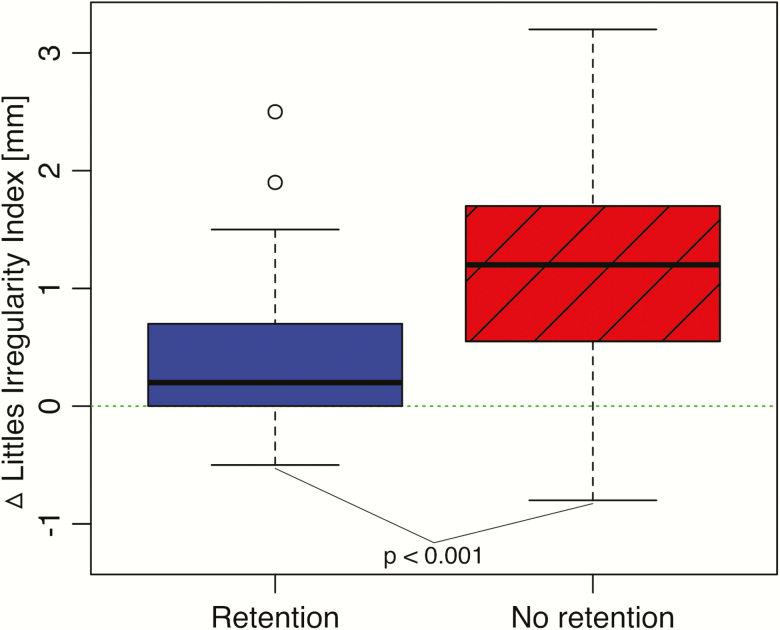
The changes in irregularity index in the two groups between T1 and T2 presented as Tukey boxplots.

The difference in irregularity change between the retention and non-retention groups was statistically significant (*P* < 0.001, 0.4 versus 1.3 mm). The maximum increase in irregularity was 2.5 mm in the retention and 3.2 mm in the non-retention group (*P* < 0.001) (Table 2; Figure 4). Moreover, the mean maximum single CPD was lower in the retention group compared to the non-retention group, but not significant (Table 2).

**Table 2.** Mean changes in mm CPD (contact point discrepancy) during the retention period (T1 = post-treatment, to T2 = 1-year follow-up) in retention group and in non-retention group. CI, confidence interval; ITT, intention-to-treat.

		Retention group (*n* = 32)				Non-retention group (*n* = 31)		
			95% CI				95% CI	
		Mean	Lower	Upper	←*P*→	Mean	Lower	Upper
Change T2 − T1	Little’s index	0.4 mm	0.2	0.6	<0.001	1.3 mm	0.9	1.7
	Arch length	0.0 mm	−0.2	0.2	0.145	−0.3 mm	−0.5	−0.1
	Intercanine width	0.1 mm	−0.1	0.3	0.279	0.4 mm	0.0	0.8
	Intermolar width	−0.1 mm	−0.3	0.1	0.492	0.0 mm	−0.4	0.4
Maximum CPD T1		0.5 mm	0.3	0.7	0.330	0.5 mm	0.3	0.7
Maximum CPD T2		0.7 mm	0.5	0.9	0.002	1.1 mm	0.9	1.3
Maximum derotation T2 − T1		4.7°	3.5	5.9	0.034	6.4°	5.4	7.4

Results (ITT) represented by means and 95% CIs.

Small but not statistically significant changes between the groups were found in arch length, intercanine and intermolar width.

**Figure 5.**
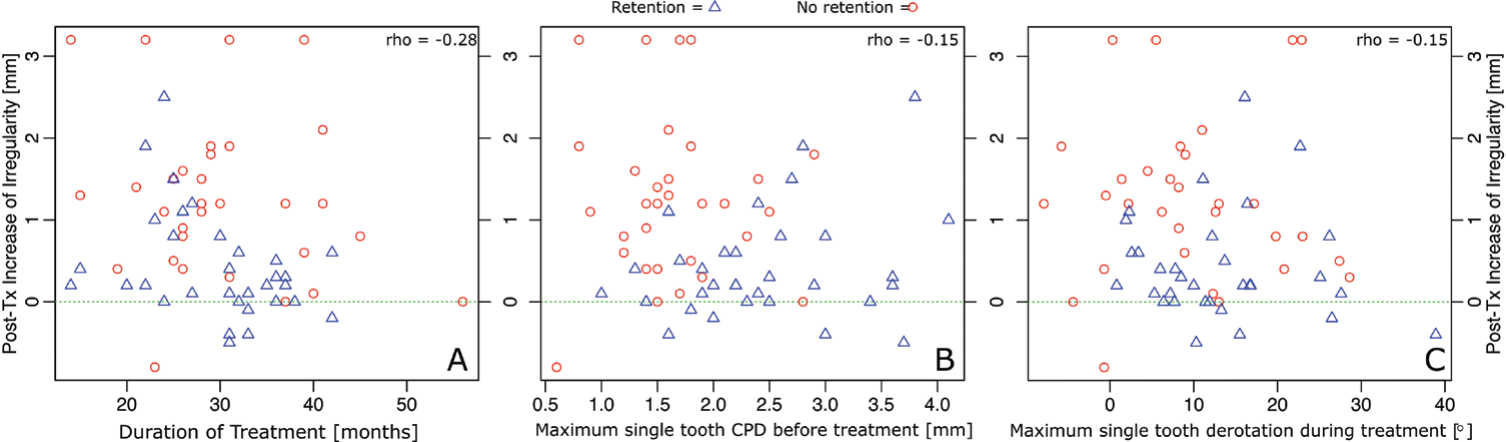
Correlations between changes in irregularity and (A) duration of treatment (rho = 0.28); (B) maximum CPD (rho = 0.15) and (C) maximum derotation (rho = 0.15). CPD, contact point discrepancy.

**Figure 6.**
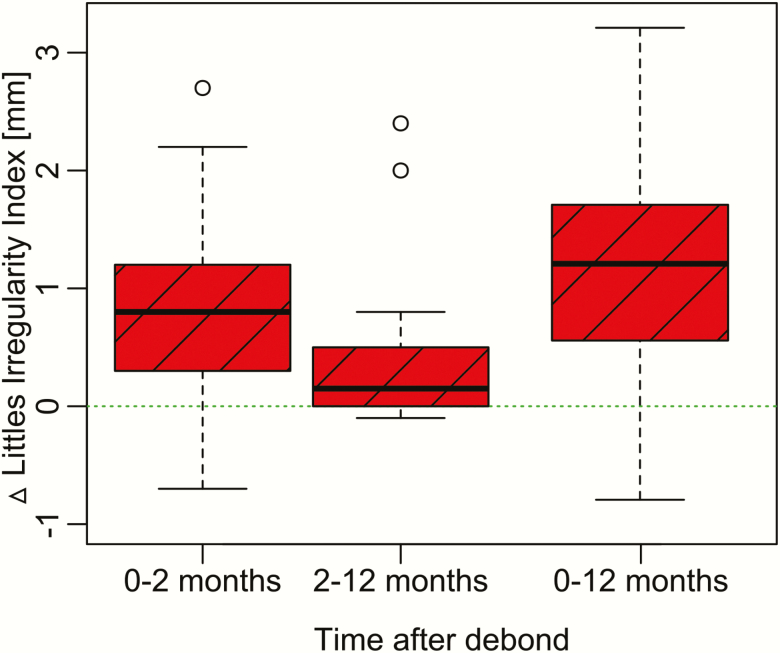
Post-treatment changes in irregularity without retention.

No significant correlations between post-treatment changes in irregularity and duration of treatment, pre-treatment CPDs or tooth derotations were detected (Figure 5). In the non-retention group, including the 10-week interim period, most changes occurred during the 10-week period (Figure 6).

In the retention group, 93.5 per cent had a LI less than 3 mm and in the non-retention group 80 per cent had a LI less than 3 mm after the 1-year observation period.

### Harms

One patient met the stopping guideline criteria. For this patient, the arch-wire was reinserted and after 2 months of realignment, the patient received a retention appliance.

## Discussion

### Main findings

**Figure 7.**
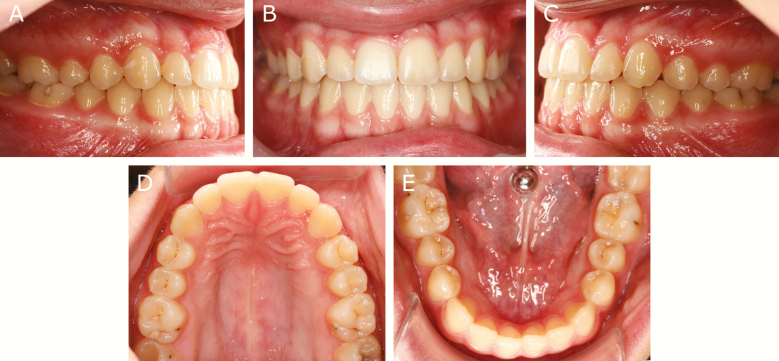
Case with Little’s index 1.9 mm.

In this unique trial, satisfactory clinical results 1-year post-treatment were found in the group without retention. Even though the average difference in LI, 0.4 versus 1.3 mm, was statistically significant, it is our opinion that this difference was not clinically significant since irregularities of LI between 1 and 3 mm are scored as minimal irregularities (Figure 7). However, it cannot be overlooked that any individual patient may have been disturbed by an irregularity of 3 mm. Consequently, our initial hypothesis could be partly confirmed and it has to be pointed out that the range of changes in the irregularity index was greater in the non-retention than in the retention group.

The challenge is to identify the patients with a higher risk for an increase in irregularity index beforehand. Since the parameters for this identification still are not entirely known, the use of the 10-week interim period in the non-retention group was useful in identifying those patients who might have a relapse immediately after treatment. Also, for ethical reasons, this interim method was justified to avoid major relapse or harm to patients. Moreover, from a short-term perspective, this trial also showed that retention with a removable vacuum-formed retainer was successful in patients with impacted maxillary canines and moderate pre-treatment dental irregularities.

The results of present trial are similar to trials that have investigated post-treatment changes of mandibular anterior teeth. Hence, these trials have reported stable results after IPR and without any retention appliances (13, 16). Regardless, it is important to admit that one patient in our trial had to be retreated for 2 months as a significant relapse was detected during the interim period. Studying the characteristics of the outliers and further investigating possible predictors of relapse may help us to identify those patients who might undergo relapse immediately after treatment.

It can also be pointed out that in the group without retention, the largest irregularity changes occurred during the first 10 weeks. These findings are in line with the results by Reitan *et al.* (30) who reported changes as early as the first day and a progressive relapse during the following 232 days until the dental fibres were re-arranged.

### Interpretations

Assessment of post-treatment changes was carried out by study casts being scanned into digital 3D models. Measuring CPDs can be considered to be a standardized task when measuring both study casts and digital 3D models. Digital 3D models are superior when it comes to measuring rotations, comparison of changes between two models or documentation of the measurements. Nevertheless, if manually placed landmarks are used it is necessary to accept a measurement error that ranges between 0.1 and 0.2 mm even in digital 3D measurements (31). Since the LI is a summary score of five contact points, the measurement error can be five times higher. The semi-automatic segmentation in OnyxCeph^3^™ (v3.2) showed a considerable variety in landmark positioning when repeated measurements were conducted, and, therefore, to avoid incorrect and inconsistent measurements the semi-automatic segmentation method was used together with manual corrections.

Owing to the anatomy of the maxillary anterior teeth the risk of incorrect measurement of CPDs is significant. However, a CPD is not always visible clinically, and a LI of less than 1 mm is often considered to be a perfect alignment. The use of a threshold value might be of interest to reduce the impact of non-visible CPDs, but a threshold value leads to a distinct partitioning of the measurements, thus introducing additional sources of errors. Therefore, in this trial no threshold values were used.

### Strengths

The dropout rate was very low in this trial and ensured comparability of both groups and satisfactory power. Moreover, the use of the ITT approach guaranteed that all patients who were randomized remained in their allocated group. Furthermore, patients with discontinued observation or lost to follow-up were regarded as failures, and, thus, the group’s maximum value for change of tooth irregularity (the primary outcome) was recorded. Consequently, the risk of false-positive treatment results was minimal in this trial. In addition, the measurement errors were regarded as small and clinically irrelevant.

Finally, the RCT methodology reduced the risk of selection bias and confounding variables were avoided by ensuring that both known and unknown determinants of outcome were uniformly allocated between the two patient groups.

### Limitations

This trial was a single-centre RCT, and the treatments were conducted by one operator. Therefore, operator-related errors might have influenced the results, but the single-centre design has the advantage of clear communication and less variance of trial conduct.

Due to the trial’s nature, blinding of the participant or care provider was not possible, which might be considered a source of bias. However, study casts were anonymized before analysis of the changes in the six maxillary anterior teeth and, in addition, the assessor had no prior involvement in the investigation.

The strength of this trial might have been further enhanced if it had included a long-term follow-up period and collection of patient’s perceptions. The need for a further long-term follow-up trial was identified and has been commenced. Nonetheless, the primary objective of this trial was to short-term assess whether retention is necessary after orthodontic treatment of impacted maxillary canines.

## Conclusions

Changes between the retention and the non-retention group were statistically but not clinically significant and since satisfactory clinical results 1-year post-treatment were found in the non-retention group retention does not appear to be needed.

Most of the relapses in the non-retention group occurred during the 10-week post-treatment interim period, and, thus, this period was useful in identifying patients who might have a relapse immediately after treatment.
